# Efficacy of Treadmill Training on Balance and Fall Risks in the Elderly: A Systematic Review."

**DOI:** 10.12688/f1000research.146583.2

**Published:** 2025-02-10

**Authors:** Ayman Mohammed Ismail Zafer, Alsayed Abdelhameed Shanb, Matar AbduAllah Alzahrani, Ankita Sharma, Moattar Raza Rizvi

**Affiliations:** 1 Physical Therapy Department, Taibah University, Medina, Al Madinah Province, 41411, Saudi Arabia; 2 Physical Therapy Department, Imam Abdulrahman Bin Faisal University, Dammam, Eastern Province, 34212, Saudi Arabia; 3 Department of Physiotherapy, School of Allied Health Sciences, Manav Rachna International Institute of Research and Studies, Faridabad, Haryana, 121004, India

**Keywords:** Elderly, balance, treadmill training, falls prevention, aging

## Abstract

**Background & Purpose:**

Falls and balance issues are significant concerns for the elderly. Treadmill training is increasingly recognized as a potential intervention to improve balance and reduce fall risk in this population. This systematic review evaluates the effectiveness of treadmill training on balance in the elderly.

**Methods:**

A comprehensive search was conducted in databases including MEDLINE, EMBASE, CINAHL Plus, PEDro, Cochrane Library, and ERIC from January 1, 1980, to January 1, 2025. The search focused on treadmill training’s impact on balance in older adults. From 74 identified studies, primary outcome measure was “Balance Improvement, risk of fall” and secondary were “Cognitive Function and Quality of Life,” and others. Articles were excluded for reasons like irrelevance to treadmill training, language barriers, or duplication, resulting in 16 final studies.

**Results:**

Treadmill training demonstrated positive effects on balance and fall risk reduction, particularly in elderly individuals with Parkinson’s disease, spinal cord injuries, and age-related balance issues. Perturbation-based treadmill training significantly reduced fall rates, while underwater treadmill sessions improved mobility and cognitive function.

**Conclusion:**

Treadmill training interventions are promising for improving balance and mobility in the elderly, including those with Parkinson’s disease, spinal cord injuries, and age-related balance issues. While these interventions show potential, further research is needed to determine optimal protocols, durations, and combinations with other interventions. This could lead to a more comprehensive approach for addressing balance and mobility challenges in the elderly, promoting healthy aging and reducing fall risks.

## Background

As the global population of older adults and elderly individuals continues to rise, there is a growing emphasis on innovative approaches to enhance their well-being, with a particular focus on addressing the challenges of aging. The aging population presents a unique set of opportunities and challenges, and by embracing innovative solutions, we can transform the aging experience, prolong vitality, and usher in a new era of longevity and well-being ( Rudnicka et al., 2020; Amuthavalli Thiyagarajan et al., 2022; Officer and de la Fuente-Núñez, 2018). Some key areas of innovation in addressing the challenges of aging include genetic therapies and stem cell regeneration, which hold great potential for improving health and function in older adults, potentially reversing age-related declines and promoting a more youthful state ( Garay, 2023; Negredo et al., 2020). Additionally, artificial intelligence and wearable technology can be used to monitor and manage health, providing valuable data for healthcare professionals and individuals to make informed decisions about their well-being ( Wang and Hsu, 2023; Bohr and Memarzadeh, 2020; Bajwa et al., 2021). Virtual reality technology offers a wealth of opportunities for the aging population, enabling them to engage in stimulating activities, travel to distant places, and socialize with others, regardless of physical limitations, thus promoting mental well-being and combating feelings of isolation and loneliness ( Chaze et al., 2022; Lin et al., 2018). Aging in place strategies focus on enhancing the safety and quality of life for older adults in their home environment, allowing them to participate in valued activities and maintain their independence.

One of the most promising areas of development is the field of exercise interventions, notably treadmill training. This approach is gaining traction as a means to counteract the age-related declines in physical function, balance, and mobility that significantly influence the quality of life and independence of older individuals ( Brach and VanSwearingen, 2013; VanSwearingen et al., 2011). Treadmill training for the elderly is particularly advantageous because it can be tailored to individual needs and physical capabilities. It offers a safe and controlled environment for exercise, which is crucial for those with diminished balance or mobility. Regular use of the treadmill can lead to improvements in balance and coordination, thereby reducing the risk of falls, a common and serious concern among the elderly ( Oddsson et al., 2007). Additionally, treadmill exercises are beneficial for cardiovascular health, helping to keep the heart healthy and potentially reducing the risk of heart-related diseases, which are more common in older age ( Chen et al., 2022; Tian and Meng, 2019; Pinckard et al., 2019; Rebelo-Marques et al., 2018). Moreover, engaging in treadmill workouts can help maintain or increase muscle strength and bone density, countering the natural decline that accompanies aging. This type of physical activity is also associated with mental health benefits, including a reduction in symptoms of depression and anxiety, which are significant issues for many older adults ( Izquierdo et al., 2021; Zhu et al., 2023). Another aspect of treadmill training that makes it suitable for older populations is its customizability. Treadmill exercises can be adjusted in terms of speed, incline, and duration to match the individual’s fitness level and physical limitations. This adaptability, combined with the safety features of modern treadmills, like safety rails and emergency stop mechanisms, ensures a safe workout environment.

In addition to physical benefits, treadmill training can also offer social benefits, especially when conducted in group settings such as classes or gym environments. It provides an opportunity for social interaction and engagement, which is important for the mental health and overall well-being of older adults. Furthermore, the integration of technology in modern treadmills, including heart rate monitors and personalized training programs, allows for more tailored and monitored exercise sessions, enhancing both safety and efficacy ( Shepherd et al., 2015; Prichard and Tiggemann, 2008). However, it is critical that any exercise regimen, including treadmill training, be developed in consultation with healthcare professionals. This ensures that the exercises are not only beneficial but also safe, particularly for those with pre-existing health conditions. A personalized approach ensures that the older adults reap the maximum benefits from their exercise routines without compromising their health. The growing interest in the geriatric population’s health and well-being is evident in the expanding research on treadmill training’s potential benefits for older adults and the elderly ( McPhee et al., 2016). This research recognizes the complex interplay of physiological, neuromuscular, and psychological factors unique to this age group. These factors play a crucial role in how older adults respond to various interventions, including exercise ( de Souto Barreto et al., 2016; Paterson et al., 2007).

Treadmill training, as a focused research area, has been the subject of numerous studies that span different years, geographical locations, and demographic profiles. These studies are not just limited in scope; they encompass a broad range of objectives and outcomes. One of the key areas of investigation is the impact of treadmill training on postural stability in the elderly. This aspect is crucial because maintaining a good postural balance is fundamental to performing everyday activities and reducing the risk of falls, which are a significant health concern in this age group ( Pereira et al., 2020, 2021; Hirjaková et al., 2020).

Another important research focus is the effect of treadmill training on gait patterns ( Newell et al., 2012; Almutairi, 2023; van Ooijen et al., 2013; Wnuk et al., 2010). As people age, their walking patterns can change, often becoming slower and less steady. These changes in gait can lead to an increased risk of falls and injuries. Treadmill training can potentially help in modifying these gait patterns, leading to safer and more efficient walking. Additionally, researchers are examining how treadmill training influences the rate of falls among the elderly ( van Ooijen et al., 2016). Falls are not only a leading cause of injury in older adults but also contribute to a fear of falling, which can significantly reduce their activity levels and quality of life. By improving balance, strength, and gait through treadmill training, there is a possibility of reducing the incidence of falls, thus enhancing the overall safety and independence of the elderly ( Cadore et al., 2013; Marsh et al., 2006). Moreover, the psychological impacts of treadmill training are also a subject of study. Regular physical activity has been associated with improvements in mood, cognitive function, and general mental well-being. For the elderly, these psychological benefits are as important as the physical ones, considering the high prevalence of depression, anxiety, and cognitive decline in this age group ( Afifi et al., 2023; Dorfman et al., 2014). The diversity of these research studies reflects the multifaceted nature of aging and the need for a comprehensive approach to health and wellness in the geriatric population. By exploring various outcomes and considering the unique challenges faced by older adults, these studies contribute significantly to our understanding of how best to support the health and well-being of the elderly through targeted interventions like treadmill training.

**Figure 2.**
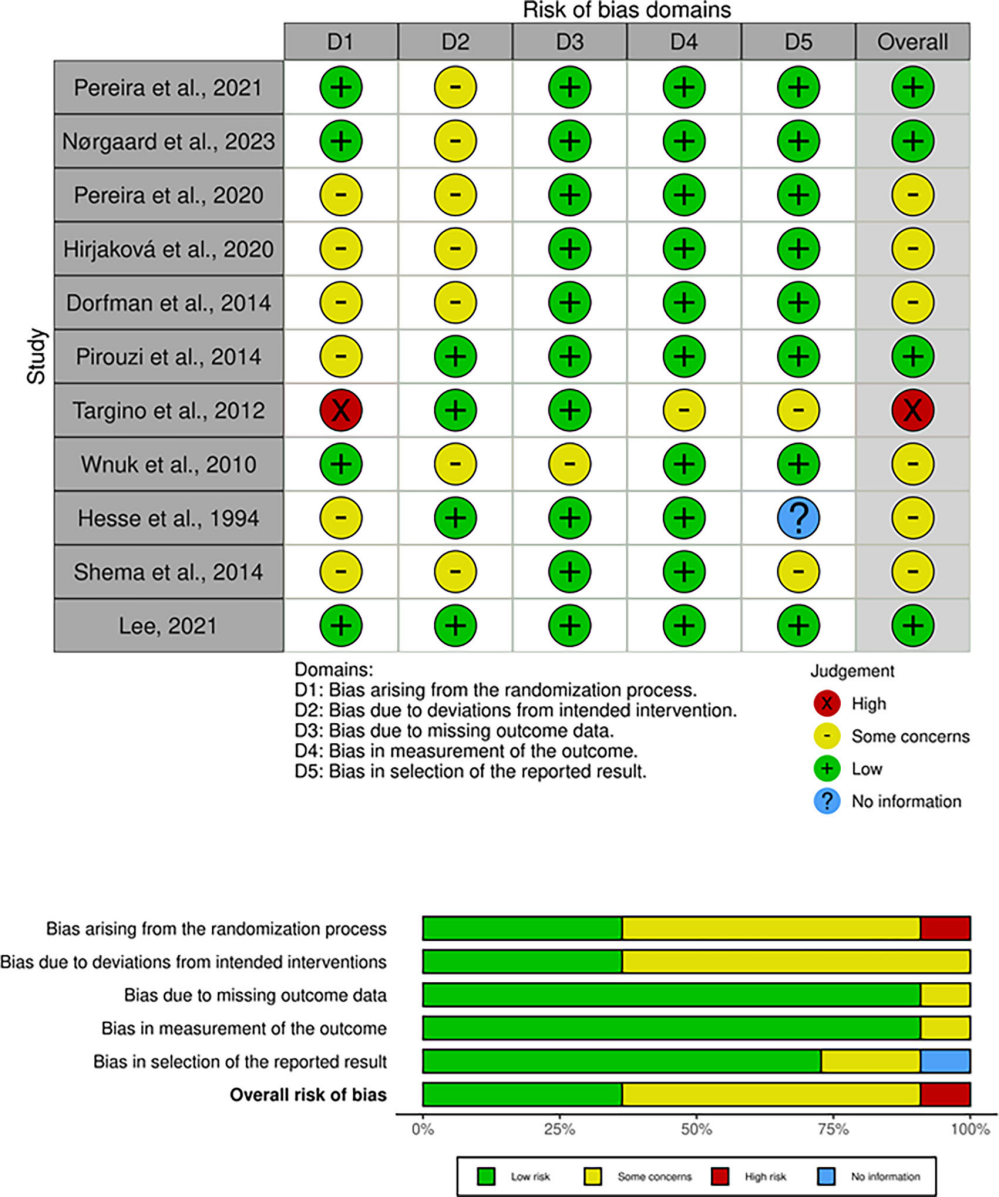
Risk of Bias Assessment. The traffic plot (top) shows domain-specific risk assessments for each study, categorized as low risk, some concerns, or high risk across five domains. The summary plot (bottom) depicts the proportion of studies at each risk level, highlighting overall methodological rigor with most studies showing low risk in outcome measurement and reporting.

The focus of this systematic review was to delve deep into the myriad of studies on treadmill training for older adults, aimed to synthesize and consolidate the diverse findings into a coherent understanding. This undertaking recognizes that understanding the mechanisms behind the observed outcomes in these studies is critical for fully realizing the potential benefits of treadmill training for the elderly. Treadmill training, with its unique ability to simultaneously engage both motor and cognitive systems, presents a promising avenue for holistic geriatric care. However, the diversity in research methodologies and outcomes necessitates a careful and comprehensive analysis to identify meaningful patterns and insights. This review, therefore, meticulously examines various aspects of the studies, including intervention protocols, study designs, and outcome measures. The goal is to identify commonalities and trends that transcend individual studies, thereby providing a more comprehensive understanding of treadmill training’s role in geriatric healthcare.

A crucial aspect of this review is the acknowledgment of the intricate relationship between physical activity and cognitive function in aging. The cognitive benefits of physical exercise, particularly in an aging population, are an area of growing interest. Treadmill training, by demanding both physical and cognitive engagement, could potentially enhance cognitive function alongside physical health, offering dual benefits to the elderly. Furthermore, this review traverses the historical landscape of treadmill training research, encompassing a broad spectrum of studies that have investigated its physiological, psychological, and social impacts. These studies have been conducted over a range of years and across various geographical and demographic contexts, adding to the richness and diversity of the data. By examining these studies, the review aims to unravel not only the direct impacts of treadmill interventions on aspects such as balance, gait, and fall risk but also their broader implications for promoting active aging, sustainable geriatric care, and community engagement.

By providing a comprehensive synthesis of these studies, this systematic review aspires to inform the development of targeted treadmill training interventions that are specifically designed to meet the needs of older adults. These interventions could potentially improve balance, reduce the risk of falls, and promote overall health and well-being in the elderly population. The ultimate goal of this endeavor is to contribute to a more nuanced and holistic approach to geriatric care, one that recognizes and utilizes the multifaceted benefits of treadmill training to enhance the quality of life for older adults and the elderly. The insights gleaned from this review could have significant implications for healthcare providers, policymakers, and caregivers, guiding them in making informed decisions about incorporating treadmill training into comprehensive care plans for the elderly.

In this systematic review, we are setting out to explore several key aspects that are critical to understanding the full scope and impact of treadmill training on older adults. These aspects are designed to provide a comprehensive overview of the subject, addressing the nuances and complexities inherent in geriatric care and exercise interventions.

1. **Types of interventions**: A crucial part of this review is the analysis of different treadmill training protocols. The aim here is to discern which specific elements of these protocols are most effective in improving balance in older adults. This analysis will consider various factors, such as the intensity, duration, and frequency of exercise, as well as the incorporation of additional equipment like body weight support systems. The goal is to identify the most beneficial practices that can be applied broadly to improve balance and reduce fall risks among the elderly.
2. **Population heterogeneity**: Recognizing that the older adult population is extremely diverse, the review will pay special attention to how different subgroups respond to treadmill training. This includes examining the experiences of individuals with varying levels of physical fitness, health conditions, and cognitive abilities. A key question is whether treadmill training is more effective or beneficial for certain subgroups, such as those with preexisting medical conditions or varying degrees of mobility impairment. Understanding these differences is crucial for tailoring interventions to meet the specific needs of different segments within the elderly population.
3. **Outcome measures**: The review rigorously evaluated a wide range of outcome measures to capture the full impact of treadmill training. This includes evaluating both static and dynamic balance, changes in gait parameters, overall mobility, incidence of falls, and improvements in the quality of life. By analyzing these diverse outcomes, the review aims to construct a holistic picture of how treadmill training affects various aspects of an elderly individual’s health and daily living.
4. **Methodological rigor**: Assessing the methodological quality of the studies included in the review is essential to determine the strength and reliability of the evidence. This involves examining each study for potential biases, methodological flaws, and limitations. This critical evaluation will provide insights into how trustworthy and applicable the results are, and will highlight areas where further research may be needed to confirm findings or fill in gaps in the current understanding.

### Inclusion and exclusion criteria

To ensure the systematic review’s primary focus on investigating the effectiveness of treadmill training in enhancing balance among the elderly population, a rigorous set of inclusion and exclusion criteria was meticulously crafted. The ensuing inclusion criteria were judiciously employed to discerningly choose studies for comprehensive analysis:

### Inclusion criteria

The review concentrated on studies encompassing individuals aged 60 years and above, thereby ensuring a dedicated examination of the elderly population. The central theme was the investigation of treadmill training interventions and their potential impact on balance improvement within this demographic. The selection criteria demanded that studies exclusively present quantitative outcomes germane to balance assessment. To attain a comprehensive perspective, the review encompassed an array of study designs, ranging from rigorous randomized controlled trials (RCTs) and non-randomized trials to observational designs such as cohort studies, cross-sectional studies, and case-control studies. The scope of the review was confined to studies published in the English language.

### Exclusion criteria

In a bid to maintain precision, studies targeting populations other than the elderly, such as individuals aged below 60, were deliberately excluded from the review. Likewise, studies appraising interventions beyond treadmill training for balance enhancement were not deemed pertinent for inclusion. Studies that failed to provide pertinent outcomes related to balance assessment were excluded to uphold the review’s integrity. Editorial pieces, opinion articles, systematic reviews, and meta-analyses were meticulously excluded to keep the focus on primary research. The purview of the review deliberately excluded abstracts, dissertations, and conference presentations, ensuring the consideration of only comprehensive research reports. Studies solely scrutinizing within-session effects of varying walking conditions were purposely omitted. Furthermore, review articles straying from the core subject matter—treadmill training’s role in improving balance among the elderly—were scrupulously excluded.

The systematic review purposefully excluded studies solely concentrating on within-session effects of different walking conditions to offer a more comprehensive view of treadmill training’s sustained efficacy. Additionally, review articles that did not align with the specific outcomes of interest—namely, the influence of treadmill training and body weight supported gait training on balance improvement among the elderly—were meticulously excluded to uphold research relevance.

Furthermore, the review remained constrained to studies accessible via peer-reviewed journals, containing full-text versions in the English language. It embraced research encompassing diverse study designs, irrespective of the approach employed. However, studies presented solely as abstracts or dissertations were intentionally omitted from consideration. Likewise, studies narrowly probing the within-session effects of diverse walking conditions were deliberately omitted. Finally, review articles addressing tangential subjects were deliberately excluded.

By diligently implementing these stringent inclusion and exclusion criteria, the systematic review aimed to curate a selection of studies closely aligned with the research objectives, eliminating those deviating from the predefined scope. This approach was meticulously tailored to deliver an exhaustive and precise evaluation of the impact of treadmill training on balance improvement within the elderly population.

## Search strategy

### Methods

For this systematic review, a comprehensive search strategy was meticulously developed to identify relevant studies that investigated the effectiveness of treadmill training on balance improvement in the elderly population. The following approach was employed to ensure the systematic retrieval of pertinent literature: Identification of Key Concepts: The search strategy began by identifying key concepts essential to the review’s focus: “treadmill training,” “elderly population,” and “balance improvement.” Keyword Formulation: To enhance search precision, specific keywords were formulated for each concept. These keywords were carefully selected to encompass various aspects of the concepts. Treadmill Training: “treadmill training,” “treadmill exercise,” “gait training,” “treadmill intervention” Elderly Population: “elderly,” “older adults,” “seniors,” “geriatric population” Balance Improvement: “balance improvement,” “postural stability enhancement,” “balance training,” “gait stability” Boolean Operator Combination: The formulated keywords were logically combined using Boolean operators (AND, OR) to create comprehensive search strings that captured the interrelation of the key concepts. (Treadmill Training OR Treadmill Exercise OR Gait Training OR Treadmill Intervention) AND; (Elderly OR Older Adults OR Seniors OR Geriatric Population) AND; (Balance Improvement OR Postural Stability Enhancement OR Balance Training OR Gait Stability); Inclusion of Controlled Vocabulary: To enhance the search strategy’s robustness, controlled vocabulary terms (MeSH terms in PubMed) were incorporated alongside the keywords to account for database-specific indexing; (“Exercise Therapy”[Mesh] OR “Gait”[Mesh] OR “Postural Balance”[Mesh]) AND; (“Aged”[Mesh] OR “Aged, 80 and over”[Mesh] OR “Elderly”[Mesh]) AND; (“Treatment Outcome”[Mesh] OR “Rehabilitation”[Mesh] OR “Physical Therapy Modalities”[Mesh]).

### Adapting the strategy for different databases

**Figure 1.**
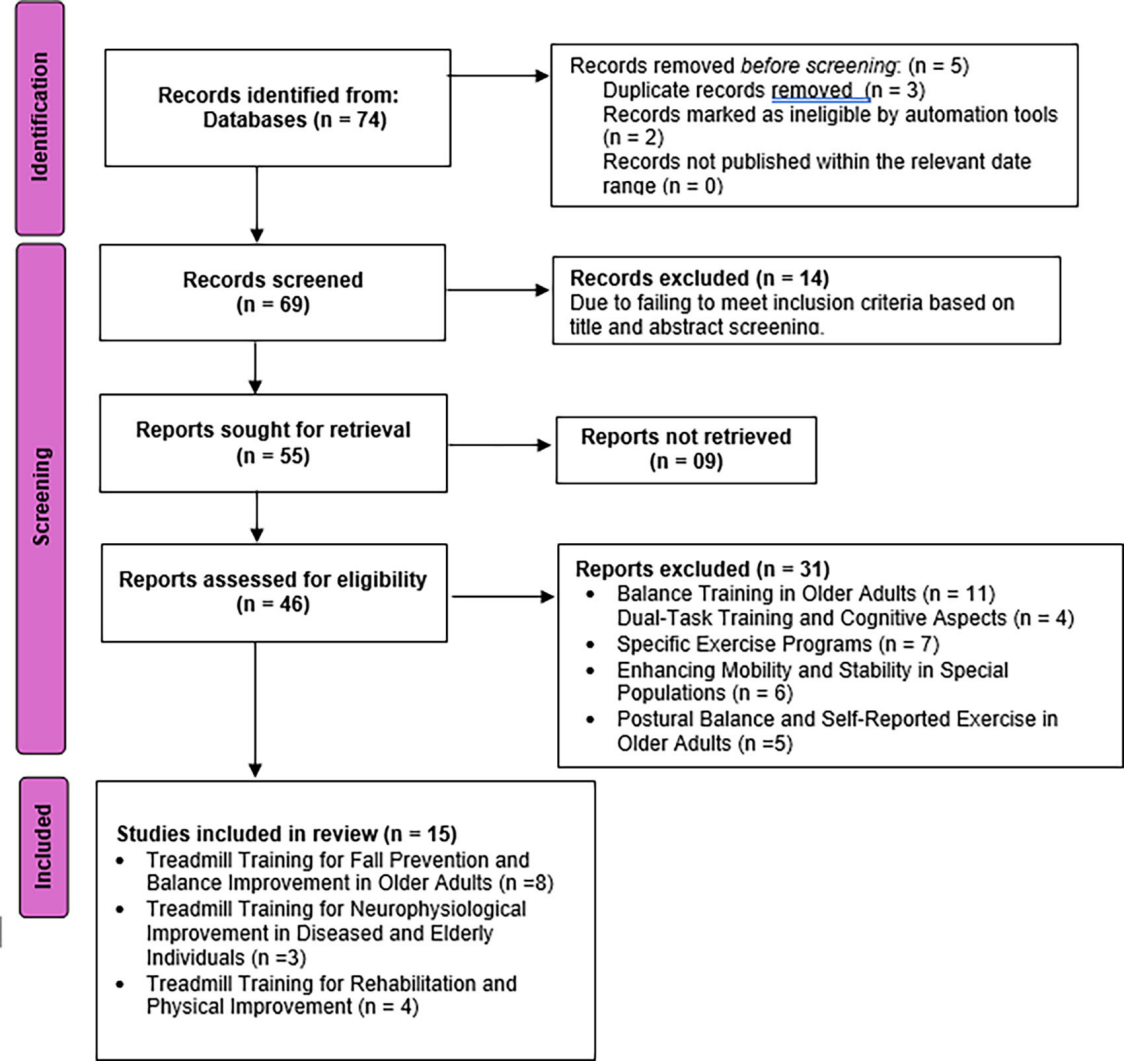
PRISMA flow chart: database search (January 1980 to May 2023).

The search strategy was tailored for various databases, ensuring compatibility with their syntax and controlled vocabulary. For Embase, Emtree terms like “Motor Activity” and “Gait” were incorporated. In CINAHL, CINAHL Headings were utilized in conjunction with relevant keywords, catering to the nursing and allied health literature. The Cochrane Library was targeted for systematic reviews and clinical trials on treadmill training and balance improvement in the elderly. Additionally, for Scopus, a combination of database-specific keywords and controlled vocabulary was utilized to achieve a comprehensive retrieval of relevant articles ( Figure 1).

### Finalizing the search strategy

This meticulous approach to constructing the search strategy aimed to systematically retrieve articles relevant to the systematic review’s objectives. By adapting the strategy for each database’s unique characteristics, it ensured the comprehensive capture of pertinent literature on the effectiveness of treadmill training on balance improvement in the elderly population.

Data were extracted independently by two reviewers using a structured data extraction form. Extracted variables included study design, sample size, participant characteristics, treadmill training protocol (duration, intensity, frequency), primary and secondary outcome measures, and key findings. Discrepancies between reviewers were resolved through discussion. After importing search results from multiple databases, duplicates were identified and removed by adding all the data in Microsoft excel, highlighting the duplicates and by manual screening. Risk of bias for each included study was evaluated using the Cochrane Risk of Bias 2.0 tool for randomized controlled trials and the Newcastle-Ottawa Scale for observational studies. The Cochrane RoB 2.0 tool assessed five domains, including bias arising from the randomization process, deviations from intended interventions, missing outcome data, measurement of the outcome, and selection of the reported result. Each domain was rated as low risk, some concerns, or high risk, with an overall rating assigned to each study. To enhance clarity, a traffic plot and a summary plot were generated to visually present the findings.

## Results

The modern landscape of healthcare and well-being is undergoing a profound transformation, propelled by the ever-expanding demographic of older adults and elderly individuals. In the pursuit of enhancing their quality of life, sustaining mobility, and mitigating age-related challenges, novel interventions are being explored with vigor. Amid this backdrop, our intention was to embark on a comprehensive exploration, elucidating the multifaceted effects of treadmill training interventions within this burgeoning demographic. The primary impetus behind curating this systematic review was to distil the collective wisdom embedded within a spectrum of studies that have investigated the effects of treadmill training in older adults and the elderly.

The selected studies, spanning from 1997 to 2023, offered an opportunity to traverse the evolutionary journey of research in this domain, and glean insights that could potentially reshape the landscape of geriatric care and rehabilitation. Through an exhaustive synthesis of these studies, our aim was to unravel a mosaic of outcomes that extended beyond the immediate purview of each investigation. The intention was to amalgamate these findings into a cohesive narrative, one that transcended individual studies and illuminated broader patterns.

The risk of bias analysis, summarized in Figure 2 (traffic plot and summary plot), revealed that 36% of studies had a low overall risk of bias, 55% exhibited some concerns, and 9% were classified as high risk. Domain-specific analysis indicated that 91% of studies demonstrated low risk in the measurement of outcomes, while 82% showed low risk in the selection of reported results. However, concerns were noted in the randomization process (45%) and deviations from intended interventions (55%). Notably, Targino et al. (2012) was rated as high risk due to significant issues in randomization and reporting. Figure X illustrates the domain-wise risk of bias for individual studies (traffic plot), while Figure Y highlights the proportion of studies classified under each risk level across the five domains assessed (summary plot). These results provide context for interpreting the reliability of the findings presented in this review.

This endeavor sought to underscore the transformative potential of treadmill training in older populations, tapping into its efficacy in restoring balance, fostering motor recovery, enhancing mobility, and ultimately elevating the overall quality of life. This systematic review engenders an in-depth exploration of the myriad effects encompassed by treadmill training interventions within the realm of older adults and elderly individuals. These studies collectively illuminate a multifaceted tapestry of outcomes that hold the potential to reshape mobility, balance, neurological well-being, and overall quality of life in this demographic ( Table 1).

**Table 1.** Studies focusing on treadmill training on elderly population.

Author(s), year	Study design	Participant characteristics	Age	Intervention	Primary outcomes	Secondary outcomes	Main findings
Treadmill Training for Fall Prevention and Balance Improvement in Older Adults:							
Effect of treadmill Perturbation-Based Balance Training on Fall Rates in Community-Dwelling Older Adults ( Nørgaard et al., 2023)	RCT	140 people aged 72 [5] years, divided into a PBT group and a control group	65 years and above	Perturbation-based balance training treadmill intervention, 4 sessions were given for 20 mins within 6 months	12-Month Fall Rates	Fall and recurrent falls, time to first fall, fall-related fractures, fall-related injuries, fall-related health care contacts, and daily-life slip and trip falls	Significant reduction in 12-month fall rates and injurious falls
Improvement of quality of life and postural balance of institutionalized elderly people undergoing to a treadmill walking training ( Pereira et al., 2021)	RCT	Intervention group (n = 23) and control group (n = 14)	60 years and older	Treadmill walking program, twice a week for 10 weeks	Postural Balance using 6-minute walk test and 10 m walk test	Quality of Life	Positive effects on postural balance and quality of life
Effectiveness of a treadmill Training Programme in Improving the Postural Balance on Institutionalized Older Adults ( Pereira et al., 2020)	RCT	Intervention group (n = 23) and control group (n = 14)	60 years and older	Treadmill walking program, twice a week for 10 weeks	Berg Balance Scale, Short Physical Performance Battery, Gait Speed	Timed Up and Go Test	Positive effects on postural balance
Postural stability after treadmill and overground walking in young and elderly ( Hirjaková et al., 2020)	Not specified	Healthy young and elderly subjects	Young adults 20-30 years, older adults 60 years and above	Treadmill and overground walking, one time	Velocity Parameters	None	Elderly became more unstable after treadmill walking, vision contributed to posture stabilization
Effects of treadmill training on gait of elders with Parkinson's disease:a literature review (2020) ( Luna et al., 2020)	Review	Patients with Parkinson's disease		Treadmill training	Gait	Nil	Positive effects on gait, can be combined with other therapies
Dual-Task Training on a Treadmill to Improve Gait and Cognitive Function in Elderly Idiopathic Fallers ( Dorfman et al., 2014)	RCT	Participant age: 78.1 ± 5.81 years, 7 women	69-85 years	Treadmill training (TT) while performing dual tasks, 3 times a week for more than 6 weeks	Berg Balance Scale, Dynamic Gait Index, gait speed	Cognitive performance, quality of life	After 6 weeks of TT + DT program, elderly fallers demonstrated improved scores on tests of mobility, functional performance tasks, and cognition. Dual task training can be readily implemented by therapists as a component of a fall-risk reduction training program
Effectiveness of Treadmill Training on Balance Control in Elderly People: A Randomized Controlled Clinical Trial ( Pirouzi et al., 2014)	RCT	30 elderly humans divided into control and experimental groups	60 years and above	Treadmill walking, 4 weeks	Gait Speed **,** Balance Ability	Postural sway	Forward and backward treadmill walk are effective in balance improvement in elderly people. Significant improvements in quiet standing on firm and foam surfaces, reduction in COP velocity during quiet standing and standing on foam
Effects of a dual-task training on dynamic and static balance control of pre-frail elderly: a pilot study ( Targino et al., 2012)	Pilot Study	Control and intervention group, Pre-frail adults	Not defined	Dual task treadmill training, 4 Weeks	Postural Control, Balance In Different Task, Static Balance	Gait	Dual-task training on treadmill improves static and dynamic balance in pre-frail elderly women. Visual stimulation aids in short-term balance maintenance
Treadmill Training for Neurophysiological Improvement in Diseased and Elderly Individuals:							
Treadmill Applications on the Neurophysiology of the Diseased and Elderly: A Review ( Morency et al., 2017)	Review	Diseased and elderly		Treadmill use	Neuroprotective Factors, Complications, Cognitive Changes	Quality of Life	Treadmill use can create neuroprotective factors, improve complications, and increase quality of life
Effects of physiotherapy including various forms of gait exercises on a treadmill on functional efficiency in the elderly at risk of falling ( Wnuk et al., 2010)	RCT	Elderly at risk of falling	60-80 Years	Gait exercises on a treadmill including backward training	Functional Efficiency	Muscle strength	Treadmill training improved functional efficiency in elderly at risk of falling
Clinical research Effect of treadmill-based gait training on the stationary balance of elderly individuals ( Monteiro et al., 2009)	RCT	60 elderly women and 60 young adult women	60 to 70 years (elderly group) or between 20 to 30 years (young adults)	Treadmill-based gait training	Center of Pressure Displacement Velocity, Radial Displacement of Center of Pressure	Not specified	Treadmill-based gait training improved stationary balance in elderly women
Treadmill Training for Rehabilitation and Physical Improvement:							
Effect of Locomotor Training on Motor Recovery and Walking Ability in Patients with Incomplete Spinal Cord Injury: A Case Series ( Anwer et al., 2014)	Case series	Adults with incomplete spinal cord injuries		Treadmill training	Motor recovery, walking ability		Improved motor recovery and walking ability
Treadmill Training with Partial Body Weight Support: Influence of Body Weight Release on the Gait of Hemiparetic Patients ( Hesse et al., 1997)	Quasi-experimental study	Six hemiparetic patients	50-72 years	Treadmill training with partial body weight support	Gait ability, motor function, gait cycle	Spasticity, muscle tone	Body weight release had significant effects on gait parameters
Clinical Experience Using a 5-Week Treadmill Training Program With Virtual Reality to Enhance Gait in an Ambulatory Physical Therapy Service ( Shema et al., 2014)	RCT	60 people with a history of falls, poor mobility, or postural instability	Not specified	Treadmill training with virtual reality 3 times per week for 5 weeks	Timed “Up & Go” Test (Tug), 2 Minute Walk Test (2Mwt), 4 Square Step Test (FSST)	Not specified	Treadmill training with VR appears to be an effective and practical tool that can be applied in an outpatient physical therapy clinic. Improvements in gait, mobility, and postural control observed
Virtual Reality Gait Training to Promote Balance and Gait Among Older People: A Randomized Clinical Trial ( Lee, 2021)	RCT	56 elderly individuals who had experienced a fall divided into a VRGT group and a control group	65 years and above	virtual reality gait training (VRGT) with non motorized treadmill	Postural Balance, temporal and spatial gait parameters		VR gait training improves balance and gait ability in elderly individuals who had experienced a fall. Notable improvements in spatiotemporal gait parameters

This comprehensive exploration doesn’t merely present a mosaic of findings; it endeavors to offer a comprehensive lens through which healthcare practitioners, researchers, and policymakers can perceive the nuanced impact of treadmill interventions. By cataloging the diversity of outcomes across studies, we aimed to provide a roadmap for refining intervention protocols, optimizing parameters, and tailoring approaches to meet the diverse needs of older adults and elderly individuals. The implications of this endeavor extend beyond the realms of academia. The insights gleaned from this systematic review hold the potential to cascade into the broader spectrum of geriatric care and societal sustainability. The sustained mobility and well-being of older individuals directly impact their active engagement in their communities, the reduction of healthcare burdens, and the conservation of resources. In weaving together the threads of these studies, we lay the foundation for a sustainable future, wherein a more comprehensive approach to geriatric well-being can be nurtured.

At the forefront of these investigations lies a profound rigorously on balance enhancement, a critical factor that directly influences fall prevention and overall functional independence in aging populations. Nørgaard et al. (2023) ingeniously introduced perturbation-based balance training on a treadmill, culminating in a compelling reduction in fall rates among community-dwelling older adults. A confluence of results from Pereira et al. (2020, 2021), Pirouzi et al. (2014), and Targino et al. (2012) further amplifies this narrative, unveiling substantial improvements in postural balance and a significant upswing in the quality of life among institutionalized elderly individuals, further emphasizing the holistic impact of treadmill interventions.

The narrative extends to older individuals grappling with neurological conditions, prominently Parkinson’s disease and hemiparesis. Luna et al. (2020) masterfully unraveled the positive implications of treadmill training on gait patterns in Parkinson’s disease, shedding light on the potential for rehabilitation and functional recovery in this population. This trajectory is mirrored in the investigation by Hesse et al. (1997), which showcased the transformative power of partial body weight-supported treadmill training in hemiparetic patients, cultivating a realm of possibilities for motor recovery. The review embarks on a comparative expedition, juxtaposing treadmill training against alternative interventions. Baek et al. (2021) orchestrated an insightful exploration into dual-task treadmill training versus conventional balance exercises, revealing the nuanced superiority of dual-task treadmill training in the realm of balance enhancement and fall risk mitigation. This illuminates the potential of incorporating cognitive engagement alongside physical training, underscoring the dynamic nature of treadmill interventions ( Table 1).

Stevens et al. (2015) courageously ventured into uncharted waters, introducing underwater treadmill training in individuals with incomplete spinal cord injuries. This groundbreaking approach unveiled an alternative dimension of therapeutic intervention, unveiling the untapped potential of aquatic treadmill training in fostering recovery and enhancing physical function, an especially significant revelation in the context of spinal cord injuries. While this comprehensive synthesis lauds the affirmative outcomes of treadmill training interventions, it also casts a discerning eye on challenges that warrant consideration. Hirjaková et al. (2020) added a layer of complexity by illuminating sensory conflicts introduced by treadmill training, particularly among elderly individuals. This observation augments our understanding of potential downsides and underscores the need for a holistic approach when designing treadmill interventions. The amalgamation of these studies not only underscores the positive outcomes but also emphasizes the exigency for standardized protocols and optimal intervention parameters across diverse populations. Collectively, these studies weave a compelling narrative that posits treadmill training as a transformative catalyst, fostering improvements in postural equilibrium, dynamic gait mechanics, and the overarching quality of life for aging individuals and those navigating intricate neurological trajectories.

Furthermore, the systematic review included a diverse array of studies that explored the effects of treadmill training interventions in older adults and the elderly ( Table 1). Shema et al. (2014) delved into the realm of outpatient physical therapy, utilizing a 5-week treadmill training program with virtual reality to enhance gait in individuals with a history of falls, poor mobility, or postural instability. The results indicated notable improvements in gait, mobility, and postural control, showcasing the effectiveness of treadmill training with virtual reality. A Randomized Controlled Clinical Trial conducted by Pirouzi et al. (2014) offered insights into the impact of treadmill walking on balance control. The study, involving 30 community dwelling older adults with a Berg Balance Scale score of 36-48, highlighted significant improvements in balance, particularly in quiet standing on firm and foam surfaces, along with a reduction in Center of Pressure (COP) velocity during quiet standing and standing on foam. Furthermore, the study by Targino et al. (2012) delved into the realm of pre-frail elderly individuals through a pilot study. The investigation revealed that dual-task training on a treadmill improved static and dynamic balance, with visual stimulation aiding in short-term balance maintenance. Lastly, Kyeongjin Lee’s study in 2020 focused on the potential of virtual reality gait training (VRGT) with non-motorized treadmill to promote balance and gait among older people who had experienced falls. This randomized clinical trial involving 56 elderly individuals highlighted the effectiveness of VRGT in improving balance and gait ability, with significant enhancements observed in spatiotemporal gait parameters.

Collectively, these studies weave a compelling narrative that posits treadmill training as a transformative catalyst, fostering improvements in postural equilibrium, dynamic gait mechanics, and the overarching quality of life for aging individuals and those navigating intricate neurological trajectories.

## Discussion

The burgeoning field of healthcare and well-being, driven by the increasing population of older adults and elderly individuals, is undergoing significant transformation. As efforts intensify to enhance their quality of life, maintain mobility, and address aging-related challenges, treadmill training interventions have emerged as a focal point of interest. This systematic review synthesizes a wide range of studies to distill collective knowledge on the impact of treadmill training on this demographic. Covering research from 1997 to 2025, it offers deep insights that could potentially reshape geriatric care and rehabilitation practices.

The review highlights the transformative impact of treadmill training in geriatric populations, particularly in enhancing postural stability, improving gait mechanics, facilitating motor recovery, and increasing functional independence. Several studies included in this review underscore treadmill training’s role in significantly reducing fall risks, particularly in high-risk elderly individuals and those with neurodegenerative conditions such as Parkinson’s disease. The majority of studies (36%) demonstrated low overall risk of bias, with 91% maintaining low risk in outcome measurement, a critical domain for reliability. While some concerns were observed in randomization processes and deviations from interventions, these issues were unlikely to compromise the validity of the findings. The high proportion of low-risk studies underscores the robustness of evidence supporting treadmill training’s efficacy in improving balance and reducing fall risks in elderly populations.

Treadmill training in geriatric populations has shown transformative potential, particularly in restoring balance, facilitating motor recovery, enhancing mobility, and ultimately elevating overall quality of life. These findings align with previous studies ( van Ooijen et al., 2013; Yang et al., 2011; Herman et al., 2007; Hirjaková et al., 2020), which demonstrated improvements in gait and balance among elderly participants following treadmill training. which demonstrated significant improvements in gait mechanics, postural stability, dynamic balance, and overall functional mobility among elderly participants following treadmill training. These studies highlight treadmill training as an effective intervention in enhancing stride length, gait velocity, and neuromuscular coordination, which are critical factors in reducing fall risk and promoting independent ambulation in aging populations. Additionally, the improvements observed in these studies suggest that task-specific motor learning through treadmill-based rehabilitation can lead to better motor control, increased lower limb strength, and improved proprioception, further supporting its widespread adoption in geriatric care (Herman et al., 2007). Similarly, Monteiro (2009) observed significant improvement in stationery balance in their study group followed the treadmill-based training. The review methodology involved a comprehensive search across databases like PubMed, Scopus, and Web of Science, focusing on publications between 1997 and 2025. The selected studies had to specifically examine the effects of treadmill training on older adults, with particular emphasis on balance, mobility, and quality of life.

The key findings from this comprehensive review illuminate several critical areas of impact regarding treadmill training for older adults and elderly individuals, each noteworthy in its implications for geriatric care. Firstly, the aspect of balance enhancement has been a significant focus. Studies conducted by Newell, (2012) and Ferhi, Hamza, et al. (2023) have been pivotal in highlighting the role of treadmill training in improving postural control, gait stability and balance. This factor is particularly crucial for preventing falls, a common risk for the elderly, and maintaining functional independence in older populations. The consistent findings across these studies underscore the effectiveness of treadmill training as a practical approach to mitigate one of the most pressing concerns in geriatric health – fall prevention. Furthermore, perturbation-based treadmill training has shown to be highly effective in reactive balance control, enabling older adults to develop faster compensatory responses to sudden postural disturbances, which is critical for fall prevention. The consistent findings across these studies reinforce treadmill training as a clinically viable and evidence-based approach to addressing one of the most pressing concerns in geriatric health—fall prevention and functional independence.

Secondly, the impact of treadmill training on individuals with neurological conditions has garnered considerable attention. The research delves into the realm of neurodegenerative diseases, particularly Parkinson’s disease and hemiparesis and multiple sclerosis ( Bishnoi et al., 2022; Robinson et al., 2019; Almutairi, 2023). Their findings indicate promising prospects for functional recovery in these groups, suggesting that treadmill training could be a valuable tool in the rehabilitation of patients with neurological impairments. The implications of these findings are far-reaching, considering the complexity and the increasing prevalence of neurological conditions among the elderly. Treadmill training has been shown to enhance gait regularity, stride length, walking speed, and coordination in individuals with Parkinson’s disease, mitigating the characteristic shuffling gait, postural instability, and freezing episodes seen in these patients (Herman et al., 2007). Additionally, treadmill-based dual-task training, which integrates cognitive and motor challenges, has demonstrated improvements in executive function and working memory, indicating potential benefits beyond mobility (Robinson et al., 2019). For individuals with stroke-induced hemiparesis, treadmill training particularly body-weight-supported treadmill walking has been found to promote neuroplastic adaptations, improve motor function in the affected limbs, and enhance walking endurance (Baek et al., 2021). The repetitive, rhythmic motion of treadmill walking stimulates cortical reorganization and motor learning, facilitating functional recovery.

Lastly, the comparative efficacy of treadmill training relative to other interventions stands out as a notable finding. Previous studies provides evidence of the superiority of treadmill training over other rehabilitative strategies, especially in enhancing balance and reducing fall risks ( Gaspar and Lapão, 2021; Choi et al., 2017). Moreover, the exploration of innovative approaches like underwater treadmill training for spinal cord injury patients, as investigated by Hammill et al. (2018), sheds light on the potential of unconventional interventions ( Dolbow et al., 2016; Hammill et al., 2018). These innovative methods broaden the scope of treadmill training, offering new avenues for rehabilitation that could be particularly beneficial for individuals with unique or more severe mobility challenges.

The review also acknowledged challenges, including sensory conflicts introduced by treadmill training, a concern highlighted in the study by Hirjaková et al. (2020). These findings emphasize the need for holistic and tailored intervention strategies.

### Implications and future research

The implications of the review findings hold relevance for both clinical practice and policy considerations. The positive impact of treadmill training on postural balance and gait parameters suggests its potential as a viable intervention to reduce fall risk among older adults. Furthermore, the review underscores the importance of individualized training programs tailored to participants’ specific needs and capacities. Despite the promising results, several avenues for future research emerge. Long-term studies evaluating the sustained effects of treadmill training interventions would contribute valuable insights. Additionally, investigating the optimal dosage, intensity, and duration of training sessions could enhance the precision of recommendations for clinical implementation. Furthermore, examining the cost-effectiveness and scalability of these interventions will be essential for broader adoption.

### Strengths and limitations

One strength of this review lies in its comprehensive search strategy, encompassing a diverse range of studies. The inclusion of various types of treadmill training interventions allowed for a nuanced understanding of the benefits across different contexts. However, limitations include potential publication bias and the heterogeneity of the selected studies, which may have influenced the synthesis of results.

## Conclusion

In conclusion, the amalgamation of these studies paints a vivid tapestry that not only underscores the affirmative outcomes but also accentuates the urgency for standardized protocols and optimal intervention parameters across diverse populations. As we weave these narratives together, a compelling collective narrative emerges, one that envisions treadmill training as a transformative catalyst, fostering advancements in postural equilibrium, dynamic gait mechanics, and the overarching quality of life for aging individuals and those navigating intricate neurological trajectories. The risk of bias analysis demonstrated strong methodological rigor in most included studies, particularly in outcome measurement and reporting. These findings provide reliable evidence supporting treadmill training as an effective intervention for improving balance and reducing fall risks among the elderly. Future research should address methodological gaps, such as enhancing randomization processes and standardizing intervention protocols. The comprehensive synthesis presented here offers a panoramic view of the potential of treadmill interventions in geriatric care, laying the groundwork for a future that champions holistic well-being in older populations.

### Ethics and consent

Ethical approval and written consent were not required.

## Data Availability

Figshare: Details of Studies Included, https://doi.org/10.6084/m9.figshare.25322011.v1 ( Rizvi, 2024a). This project contains the following data:
- Details of Studies Included.xlsx Figshare: PRISMA Flow chart, https://figshare.com/account/articles/25323391?file=51629657. This project contains the following extended data:
- PRISMA Flow chart Figshare: Risk of Bias Assessment, https://figshare.com/account/items/28193594/edit This project contains the traffic plot that shows domain-specific risk assessments for each study, categorized as low risk, some concerns, or high risk across five domains and summary plot that depicts the proportion of studies at each risk level, highlighting overall methodological rigor with most studies showing low risk in outcome measurement and reporting. Fig share: PRISMA check list, https://figshare.com/account/articles/25323391?file=51629657. Data are available under the terms of the CC BY 4.0 Attribution International License (CC BY 4.0).
