# Supplementary material for: Efficacy of Treadmill Training on Balance and Fall Risks in the Elderly: A Systematic Review."
Source: F1000Res. 2025 Feb 10;13:330. Originally published 2024 Apr 23. [Version 2] doi: 10.12688/f1000research.146583.2 (PMC12344403; doi:10.12688/f1000research.146583.2)
Supplement: Peer review history

# Reviewer report: Reviewer response for version 2

**DOI:** 10.5256/f1000research.177083.r394714

**Published:** 2025-08-12

**Authors:** Hafsah Arshad

**Affiliations:** 1 Ibadat International University, Islamabad, Pakistan

This systematic review is well-executed and scientifically sound. It meets all key criteria for transparency, methodological rigor, and clinical relevance. With minor enhancements for reproducibility, this article would represent a high-quality contribution to the field.

 The findings offer meaningful insights for clinical practice, policy formulation, and future research, particularly in guiding evidence-based interventions and improving patient outcomes.

Are the rationale for, and objectives of, the Systematic Review clearly stated?

Yes

Is the statistical analysis and its interpretation appropriate?

Yes

If this is a Living Systematic Review, is the ‘living’ method appropriate and is the search schedule clearly defined and justified? (‘Living Systematic Review’ or a variation of this term should be included in the title.)

Yes

Are sufficient details of the methods and analysis provided to allow replication by others?

Yes

Are the conclusions drawn adequately supported by the results presented in the review?

Yes

Reviewer Expertise:

neurological physical therapy

I confirm that I have read this submission and believe that I have an appropriate level of expertise to confirm that it is of an acceptable scientific standard.

# Reviewer report: Reviewer response for version 2

**DOI:** 10.5256/f1000research.177083.r365510

**Published:** 2025-03-10

**Authors:** Mohamed Salaheldien Alayat

**Affiliations:** 1 Physiotherapy and rehabilitation department, Faculty of Applied Medical science, Umm Al-Qura University, Mecca, Saudi Arabia; 2 Basic Science, Cairo University, Faculty of Physical Therapy, Giza, Egypt, Egypt

The authors respond to all the reviewer comments in the revised version. Risk of bias was successfully added. I think paper now is ready for indexing.

Are the rationale for, and objectives of, the Systematic Review clearly stated?

Yes

Is the statistical analysis and its interpretation appropriate?

Partly

If this is a Living Systematic Review, is the ‘living’ method appropriate and is the search schedule clearly defined and justified? (‘Living Systematic Review’ or a variation of this term should be included in the title.)

Partly

Are sufficient details of the methods and analysis provided to allow replication by others?

Partly

Are the conclusions drawn adequately supported by the results presented in the review?

Yes

Reviewer Expertise:

Physical Therapy, Rehabilitation, Therapeutic Modalities.

I confirm that I have read this submission and believe that I have an appropriate level of expertise to confirm that it is of an acceptable scientific standard.

# Response

**Published:** 2025-03-13

**Authors:** Moattar Rizvi

**Affiliations:** Physiotherapy, Manav Rachna International Institute of Research and Studies, Faridabad, Haryana, India

Accepted

# Reviewer report: Reviewer response for version 1

**DOI:** 10.5256/f1000research.160679.r353972

**Published:** 2025-01-09

**Authors:** Adel Alhusaini

**Affiliations:** 1 Department of Health Rehabilitation Sciences, College of Applied Medical Sciences, King Saud University, Riyadh, Riyadh Province, Saudi Arabia

This systematic review offers a significant contribution to understanding the efficacy of treadmill training in enhancing balance and reducing fall risk among older adults. The methodology encompasses a comprehensive literature search and analysis of approximately fifteen studies evaluating various treadmill training protocols. The findings suggest potential benefits for improving balance and mobility, particularly for individuals with Parkinson's disease or spinal cord injuries. The conclusions appear largely substantiated by the presented evidence, and the acknowledgment of limitations is appropriate. The authors rightly emphasize the significance of synthesizing disparate research. A recommendation for further investigation to optimize training protocols and address individualized needs is critical. However, several aspects warrant further consideration to enhance the review's clarity, rigor, and practical applicability.

 Methodological Clarity and Transparency: An inconsistency in the reported number of included studies (n=16 in the abstract versus n=15 in Table 1) warrants attention regarding transparency. The authors should explicitly state the final number of studies included within the results section and reconcile this discrepancy through verification and subsequent updates to both sections.

 Data Interpretation and Contextualization: Regarding Table 1: study by Hesse et al., 1997, updating the 'Study Type' field to 'Quasi-experimental study' is recommended if methodologically accurate. Furthermore, contextualizing the findings in relation to existing systematic reviews and meta-analyses would significantly enrich the review by providing a broader scholarly perspective and identifying areas of convergence or divergence.

 Methodological Rigor and Bias Assessment: Explicit reporting of blinding procedures within the included studies is necessary to facilitate a robust assessment of potential bias. Acknowledging limitations arising from the absence or non-reporting of blinding procedures is also crucial for a comprehensive evaluation of study quality.

 Please carefully review Table 1's columns and consider these revisions for clarity and conciseness:

 Change "Paper Title" to "Author(s), Year" without the title for easier reference and table management.

 Change "Study Type" to the more precise "Study Design."

 Revise "Population Characteristics" to "Participant Characteristics" and add an "Age" field for comprehensive participant details.

 Add a "Country/Setting" column to provide important contextual information, if possible.

 Combine "Intervention" and "Duration" into a single column for better readability (e.g., "Treadmill training, 8 weeks").

 Add separate columns for "Primary Outcome(s)" and "Secondary Outcome(s)" of the studies.

 Include a column specifying the "Outcome Measurement Tools" used for each outcome.

 Furthermore, concerning citation analysis within Table 1, if a literature review is cited, it is essential to examine the original primary studies referenced within that review and include those primary studies in the systematic review if they meet the pre-defined inclusion criteria.

Are the rationale for, and objectives of, the Systematic Review clearly stated?

Yes

Is the statistical analysis and its interpretation appropriate?

Not applicable

If this is a Living Systematic Review, is the ‘living’ method appropriate and is the search schedule clearly defined and justified? (‘Living Systematic Review’ or a variation of this term should be included in the title.)

Not applicable

Are sufficient details of the methods and analysis provided to allow replication by others?

Partly

Are the conclusions drawn adequately supported by the results presented in the review?

Yes

Reviewer Expertise:

Developmental Disabilities, Early Mobility, Infant Movement, Motor Skills, Motor Control, Gait, Balance, Coordination, Assistive Technology, Childhood Obesity, Physical Function, Physical Activity, Sedentary Behavior, Participation Restrictions, ICF (Children), Outcome Measures & Assessment, Handwriting, Quality of Life (Pediatrics), Pediatric Rehabilitation, Early Intervention, Child Development, Motor Delay, Postural Control, Motivation, Self-Efficacy, Anxiety (Movement-Related), Social Participation, Family Involvement, Caregiver Burden, School Integration.

I confirm that I have read this submission and believe that I have an appropriate level of expertise to confirm that it is of an acceptable scientific standard, however I have significant reservations, as outlined above.

# Response

**Published:** 2025-01-15

**Authors:** Moattar Rizvi

**Affiliations:** Physiotherapy, Manav Rachna International Institute of Research and Studies, Faridabad, Haryana, India

**Many Thank for  prof. Adel Alhusaini  for your valuable and worthful comments**

 The responses for all reviewing comments one by one 

**1-Reviewer comment**

 Methodological Clarity and Transparency: An inconsistency in the reported number of included studies (n=16 in the abstract versus n=15 in Table 1) warrants attention regarding transparency. The authors should explicitly state the final number of studies included within the results section and reconcile this discrepancy through verification and subsequent updates to both sections.

**1-Author response** 

 Thank you for pointing out this inconsistency. Upon verification, the correct number of included studies is **15**, and the mention of **16 studies in the abstract was a typographical error**. We have now updated the abstract, results section, and any other relevant parts of the manuscript to reflect the correct count ( **n=15**) to maintain accuracy and transparency.

**2-Reviewer comment**

 Data Interpretation and Contextualization: Regarding Table 1: study by Hesse et al., 1997, updating the 'Study Type' field to 'Quasi-experimental study' is recommended if methodologically accurate. Furthermore, contextualizing the findings in relation to existing systematic reviews and meta-analyses would significantly enrich the review by providing a broader scholarly perspective and identifying areas of convergence or divergence.

 Methodological Rigor and Bias Assessment: Explicit reporting of blinding procedures within the included studies is necessary to facilitate a robust assessment of potential bias. Acknowledging limitations arising from the absence or non-reporting of blinding procedures is also crucial for a comprehensive evaluation of study quality.

**2- Author comments**

 Thank you for your suggestion. The assessment of blinding procedures has been conducted using the **Risk of Bias Tool (RoB 2 for RCTs)**. The updated **Results section** now explicitly reports blinding practices within the included studies, identifying whether **single, double, or no blinding** was used.

 Additionally, we have now included a detailed **bias assessment table** to highlight the impact of blinding (or lack thereof) on study outcomes. In cases where blinding was not reported, the potential risk of **performance and detection bias** has been acknowledged.

**3- Reviewer comment**

  Please carefully review Table 1's columns and consider these revisions for clarity and conciseness:

 Change "Paper Title" to "Author(s), Year" without the title for easier reference and table management.

 Change "Study Type" to the more precise "Study Design."

 Revise "Population Characteristics" to "Participant Characteristics" and add an "Age" field for comprehensive participant details.

 Add a "Country/Setting" column to provide important contextual information, if possible.

 Combine "Intervention" and "Duration" into a single column for better readability (e.g., "Treadmill training, 8 weeks").

 Add separate columns for "Primary Outcome(s)" and "Secondary Outcome(s)" of the studies.

 Include a column specifying the "Outcome Measurement Tools" used for each outcome

**3- Author response**

 Thank you for your detailed and valuable suggestions. We have carefully revised **Table 1** to improve clarity, conciseness, and readability in accordance with your recommendations:

- The **"Paper Title"** column has been changed to **"Author(s), Year"**, listing only the author(s) and publication year for streamlined reference.
- **"Study Type"** has been updated to **"Study Design"** to ensure precision in methodological classification.
- **"Population Characteristics"** has been revised to **"Participant Characteristics,"** and an **"Age"** field has been added to provide a clearer demographic profile of study participants.
- A new **"Country/Setting"** column has been included where possible to offer contextual insights into the study environments.
- The **"Intervention" and "Duration"** columns have been combined into a single column to improve table readability (e.g., "Treadmill training, 8 weeks").
- Separate columns for **"Primary Outcome(s)"** and **"Secondary Outcome(s)"** have been added to distinguish key findings in a structured manner..

**4-Reviewer comment**

 Furthermore, concerning citation analysis within Table 1, if a literature review is cited, it is essential to examine the original primary studies referenced within that review and include those primary studies in the systematic review if they meet the pre-defined inclusion criteria.

**4- Author response**

 Thank you for your insightful suggestion. We conducted a thorough **citation analysis** of the literature reviews included in Table 1 to identify relevant **primary studies** that could potentially meet our pre-defined inclusion criteria. However, upon careful examination, **none of the primary studies referenced within the cited literature reviews met the eligibility criteria** for inclusion in this systematic review.

**Many thanks for your efforts and worthful comments**

# Reviewer report: Reviewer response for version 1

**DOI:** 10.5256/f1000research.160679.r351604

**Published:** 2024-12-31

**Authors:** Mohamed Salaheldien Alayat

**Affiliations:** 1 Physiotherapy and rehabilitation department, Faculty of Applied Medical science, Umm Al-Qura University, Mecca, Saudi Arabia; 2 Basic Science, Cairo University, Faculty of Physical Therapy, Giza, Egypt, Egypt

Title:

 The title containing “geriatric care” and “elderly population”. Use one of them only. Suggested title” efficacy of treadmill training on balance and fall risks in elderly population. A systematic review”.

**Abstract:**

 1) Search until May 31, 2023. It seems to be old and needed for update.

 2) “From 74 identified studies, outcome measures were categorized into groups like “Balance Improvement,” “Gait Improvement,” “Mobility Enhancement,” “Muscle Strength Improvement,” “Cognitive Function and Quality of Life,” and others” No need for all alternative words for measuring balance. The main measured outcome was balance.

 3) The excluded Articles criteria are not placed in the abstract. It is better to be placed in the methodology section.

 4) The results for fall risks reduction were not mentioned in the result section in the abstract. It is your second measured outcomes as mentioned in the title and research aim.

**Introduction:**

 You used present or future tense in writing “The review will rigorously”. Please use the past tense. “ The focus of this systematic review is to delve deep”. Do not use we or our, use the third pronoun like it was …..

**Methodology:**

 1) Use the updated for of PRISMA flow chart (PRISMA 2020 flow diagram for new systematic reviews which included searches of databases, registers and other sources). The new one included the Identification of studies via databases and registers and Identification of studies via other methods.

 2) Mention the methods of identifying and removing the duplicates in different databases, the data extraction criteria, the used methods of methodological quality assessment.

 3) Why you split the table including subtitle “Treadmill Training for Neurophysiological Improvement in Diseased and Elderly Individuals”.

 4) Where the results of assessment of methodological quality in the result section.

 5) Discussion needs more extensive report of finding of this systematic review.

Are the rationale for, and objectives of, the Systematic Review clearly stated?

Yes

Is the statistical analysis and its interpretation appropriate?

Partly

If this is a Living Systematic Review, is the ‘living’ method appropriate and is the search schedule clearly defined and justified? (‘Living Systematic Review’ or a variation of this term should be included in the title.)

Partly

Are sufficient details of the methods and analysis provided to allow replication by others?

Partly

Are the conclusions drawn adequately supported by the results presented in the review?

Yes

Reviewer Expertise:

Physical Therapy, Rehabilitation, Therapeutic Modalities.

I confirm that I have read this submission and believe that I have an appropriate level of expertise to confirm that it is of an acceptable scientific standard, however I have significant reservations, as outlined above.

# Response

**Published:** 2025-01-15

**Authors:** Moattar Rizvi

**Affiliations:** Physiotherapy, Manav Rachna International Institute of Research and Studies, Faridabad, Haryana, India

Thanks prof ,Mohamed Salah for your valuable & worthful comments and your suggestions 

 The responses of author to all comments one by one 

 1-Comment1-  Title:

 The title containing “geriatric care” and “elderly population”. Use one of them only. Suggested title” efficacy of treadmill training on balance and fall risks in elderly population. A systematic review”.

 1- Author response 

 Thank you for your valuable suggestion. We have modified the title to align with your recommendation. The revised title is:

**"Efficacy of Treadmill Training on Balance and Fall Risks in the Elderly: A Systematic Review."**

**Abstract**

**1 - Reviewer Comment**

**Abstract:**

 1) Search until May 31, 2023. It seems to be old and needed for updates.

 1-Author Response 

 Thank you for pointing this out. We have updated the search to include studies published up to **January 1, 2025.** After rerunning the search using the same strategy, no additional articles matching the inclusion criteria were identified. Therefore, the original findings remain unchanged.​​​​​​​

**2- Reviewer Comment**

 2) “From 74 identified studies, outcome measures were categorized into groups like “Balance Improvement,” “Gait Improvement,” “Mobility Enhancement,” “Muscle Strength Improvement,” “Cognitive Function and Quality of Life,” and others” No need for all alternative words for measuring balance. The main measured outcome was balance.

    2 -Author response 

 Thank you for your feedback. We have revised the text to emphasize balance as the primary outcome measure while retaining the mention of secondary outcomes. The updated text now reads:

**"From 74 identified studies, the primary outcome measure was ‘Balance Improvement,’ while secondary outcomes included ‘Cognitive Function and Quality of Life,’ among others."**

 This revision ensures clarity and aligns with the focus of the systematic review

 3-Reviewer comment 

 The excluded Articles criteria are not placed in the abstract. It is better to be placed in the methodology section.

 3-Author response 

 Thank you for the suggestion. We have removed the excluded articles criteria from the abstract and incorporated them into the Methodology section.

 4-Reviewer comment 

 The results for fall risks reduction were not mentioned in the result section in the abstract. It is your second measured outcomes as mentioned in the title and research aim.

 4-Author response 

 Thank you for your valuable feedback. We have revised the abstract's results section to explicitly mention fall risk reduction. The updated text now reads:

**"Treadmill training demonstrated positive effects on balance and fall risk reduction, particularly in elderly individuals with Parkinson’s disease, spinal cord injuries, and age-related balance issues. Perturbation-based treadmill training significantly reduced fall rates, while underwater treadmill sessions improved mobility and cognitive function."**

**Introduction:**

**1-Reviewer comment**

 You used present or future tense in writing “The review will rigorously”. Please use the past tense. “ The focus of this systematic review is to delve deep”. Do not use we or our, use the third pronoun like it was

 1-Author response 

 Thank you for your insightful feedback. We have revised the text to maintain consistency in past tense and third-person narration. The updated sentences now read:

- **"The review rigorously evaluated..."** (instead of “The review will rigorously...”)
- **"The focus of this systematic review was to examine..."** (instead of “The focus of this systematic review is to delve deep...”)

**Methodology:**

 1-Reviewer comment 

 Use the updated for of PRISMA flow chart (PRISMA 2020 flow diagram for new systematic reviews which included searches of databases, registers and other sources). The new one included the Identification of studies via databases and registers and Identification of studies via other methods.

 1-Author response 

 Thank you for your valuable suggestion. We have updated the PRISMA flowchart to the **PRISMA 2020 format**, which now includes a more detailed representation of the study selection process. The revised flowchart distinguishes between **identification of studies via databases and registers** and **identification via other methods** as per the updated PRISMA guidelines.

  2-Reviewer comment 

 Mention the methods of identifying and removing the duplicates in different databases, the data extraction criteria, the used methods of methodological quality assessment.

 2-Author response 

 Thank you for your valuable feedback. We have now explicitly detailed the methods used for duplicate removal, data extraction, and quality assessment in the **Methodology section**. The revised text is as follows:

 Data were extracted independently by two reviewers using a structured data extraction form. Extracted variables included study design, sample size, participant characteristics, treadmill training protocol (duration, intensity, frequency), primary and secondary outcome measures, and key findings. Discrepancies between reviewers were resolved through discussion. After importing search results from multiple databases, duplicates were identified and removed by adding all the data in Microsoft excel, highlighting the duplicates and by manual screening. Risk of bias for each included study was evaluated using the Cochrane Risk of Bias 2.0 tool for randomized controlled trials and the Newcastle-Ottawa Scale for observational studies. The Cochrane RoB 2.0 tool assessed five domains, including bias arising from the randomization process, deviations from intended interventions, missing outcome data, measurement of the outcome, and selection of the reported result. Each domain was rated as low risk, some concerns, or high risk, with an overall rating assigned to each study. To enhance clarity, a traffic plot and a summary plot were generated to visually present the findings

 3-Reviewer comment 

 Why you split the table including subtitle “Treadmill Training for Neurophysiological Improvement in Diseased and Elderly Individuals”.

 3-Author response 

 Thank you for your feedback. The table was originally split to enhance clarity and organization by grouping studies based on their primary focus areas. The subtitle “Treadmill Training for Neurophysiological Improvement in Diseased and Elderly Individuals” was maintained to ensure a clear distinction between studies targeting neurophysiological improvements and those addressing general balance and mobility.

 However, based on your suggestion, we have revised the table format to ensure better coherence:

 Merged the table sections where appropriate to present a more unified dataset.

 Maintained logical categorization within a single table to improve readability without unnecessary fragmentation.

 Used clear subheadings instead of splitting tables to differentiate study categories while maintaining flow.

 4-Reviewer comment 

 Where the results of assessment of methodological quality in the result section.

 4-Author response 

 Thank you for your valuable feedback. We acknowledge that the assessment of methodological quality was not explicitly reported in the results section. To address this, we have now included a detailed summary of the methodological quality of the included studies.

 5-Reviewer comment 

 Discussion needs more extensive report of finding of this systematic review.

 5-Author response 

 Thank you for your valuable suggestion. We have expanded the **Discussion section** to provide a more comprehensive analysis of the key findings of this systematic review. The revised section now includes a more detailed synthesis of the results, highlighting the impact of treadmill training on balance improvement and fall risk reduction, along with its effects on secondary outcomes such as cognitive function and quality of life.

 The updated **Discussion** now explicitly contrasts the findings of included studies, addressing variations in study design, population characteristics, and intervention protocols. We have also included a comparative discussion of different treadmill training modalities, such as perturbation-based treadmill training and underwater treadmill training, emphasizing their relative effectiveness. Additionally, we have elaborated on the implications of these findings for clinical practice and rehabilitation, while also acknowledging existing research gaps and the need for future studies to establish standardized training protocols.

 ​​​​​​​Very thanks for your comments
